# Micro- and Nanoscale Topographies on Silk Regulate Gene Expression of Human Corneal Epithelial Cells

**DOI:** 10.1167/iovs.17-22213

**Published:** 2017-12

**Authors:** Kai B. Kang, Brian D. Lawrence, X. Raymond Gao, Yuncin Luo, Qiang Zhou, Aihong Liu, Victor H. Guaiquil, Mark I. Rosenblatt

**Affiliations:** 1 Department of Ophthalmology and Visual Sciences, Illinois Eye and Ear Infirmary, University of Illinois at Chicago, Chicago, Illinois, United States; 2 Department of Ophthalmology, Weill Cornell Medical College, New York, New York, United States

**Keywords:** gene expression, human cornea, silk nanotechnology

## Abstract

**Purpose:**

Corneal basement membrane has topographical features that provide biophysical cues to direct cell adherence, migration, and proliferation. In this study, we hypothesize that varying topographic pitch created on silk films can alter epithelial cell morphology, adhesion, and the genetic expression involved in cytoskeletal dynamics-related pathways.

**Methods:**

Silicon wafers with parallel ridge widths of 2000, 1000, and 800 nm were produced and used to pattern silk films via soft lithography. Human corneal epithelial cells were cultured onto silk. After 72 hours of incubation, images were taken to study cell morphology and alignment. Cytoskeletal structures were studied by immunofluorescent staining. RNA was collected from cultured cells to perform RNA-Seq transcriptome analysis using the Illumina Hiseq 2500 sequencing system. Differentially expressed genes were identified using DNAstar Qseq then verified using quantitative real-time PCR. These genes were used to perform pathway analyses using Ingenuity Pathways Analysis.

**Results:**

Primary human corneal epithelial cell alignment to the surface pattern was the greatest on 1000-nm features. Fluorescent microscopy of f-actin staining showed cell cytoskeleton alignment either in parallel (2000 nm) or perpendicular (1000 and 800 nm) to the long feature axis. Z-stack projection of vinculin staining indicated increased focal adhesion formation localized on the cellular basal surface. RNA-seq analysis revealed differentially expressed genes involved in actin organization, integrin signaling, and focal adhesion kinase signaling (−log (*P*)>5).

**Conclusions:**

Patterned silk film substrates may serve as a scaffold and provide biophysical cues to corneal epithelial cells that change their gene expression, alter cellular adherence, morphology, and may offer a promising customizable material for use in ocular surface repair.

Various traumatic insults, ocular medications, and immunological processes can cause damage to the cornea.^1,2^ Each of these processes may also cause permanent damage to the reservoir of corneal epithelial stem cells, residing at the corneal limbus, that proliferate during corneal epithelial regeneration.^3^ In order for corneal epithelial regeneration to proceed properly, the wounded tissue requires a substratum that promotes the adherence and orderly migration of the corneal epithelium. In the human eye, the corneal epithelium is anchored to the basement membrane. In addition to the biochemical features of the basement membrane, careful scanning electron microscopy (SEM) demonstrates it contains a significant number of topographical features in the 70 to 200 nm range.^4,5^ These nanoscale features may direct the ability for epithelial cells to adhere, proliferate, and migrate on their basement membrane.^4–10^ The effects of micro- and nano-patterned substrates consisting of different biomaterials on cellular function has been investigated.^6,9–11^ It was shown that various topographical features are able to provide biophysical cues to different cell types, leading to changes in cell adhesion and cell morphology.^6,9–11^ In addition, many stem cell types express high levels of various cell surface receptors that can directly sense the physical properties of the extracellular matrix (ECM) on which they reside, provide anchorage to the ECM, and elicit downstream biochemical signaling responses in the process of “mechanotransduction.”^11–16^ Pathways activated by mechanotransduction can lead to important changes in cell behavior and function.^11–16^

The silk fibroin protein has been utilized as a biomaterial for tissue engineering and regenerative medicine due to its biocompatibility, material stability, mechanical robustness, and controllable degradability.^17–21^ Silk fibers have been used as surgical sutures for centuries, and recently solubilized fibers have been used to produce regenerated silk scaffolds like sponges, hydrogels, and films.^18^ Silk films can be produced that are highly transparent in the visible light spectrum, and due to these properties, silk films are potentially suitable for ophthalmic applications in relation to tissue engineering and regenerative medicine. In addition, silk film surfaces can be easily modified through topographic patterning utilizing standard soft-lithography approaches.^22^ This allows for the design of micro- and nanoscale silk film topographies to study the effect of systematically altering the corneal epithelial cell microenvironment on cell structure and function.

Studying how the microenvironment alters cellular function allows for the identification of patterns of cellular gene expression changes that can help to reveal key genes and pathways involved in these processes. Currently, little is known in regards to how surface topography affects gene expression and gene regulation of corneal epithelial cells. Previously, our group has shown that micro-patterned silk film surfaces can guide the migratory direction of individual cells making up the collective epithelial sheet.^22,23^ However, how this occurs and the genetic basis for this phenomenon is still unknown. Our present work utilizes various silk film surface features of various pitch and width dimensions to study the response of human corneal epithelial cell cultures to topographic cues ranging from the micro- to nanoscale. Specifically, changes in cell cytoskeletal architecture and organization were observed, and then changes in gene expression were assessed respectively. Results from these studies indicate that the presence of surface topography enhances a variety of cell responses in relation to cytoskeleton dynamics.

## Materials and Methods

### Production of Silk Solution

Bombyx mori silkworm cocoons (Tajima Shoji Co., Yokohama, Japan) were cut into thirds and then boiled for 40 minutes in 0.02 M Na_2_CO_3_ (Sigma-Aldrich Corp., St. Louis, MO, USA) to extract the glue-like sericin proteins from the structural fibroin proteins as previously described.^5^ The fibroin extract was then rinsed three times in dH_2_O for 20 minutes per wash then dried overnight. The rinsed fibroin extract was then dissolved in 9.3 M LiBr solution at room temperature, and placed covered within a 60°C oven for 4 hours. The solution was dialyzed in water for 48 hours (MWCO 3500, Pierce, Inc., Thermo Scientific, Rockford, IL, USA). The dialyzed silk solution was centrifuged twice at 13,000*g*, and the supernatant collected and stored at 4°C. The final concentration of aqueous silk solution was 8 wt/vol %, as determined by gravimetric analysis.

### Flat and Patterned Silk Film Preparation

**Figure 1:**
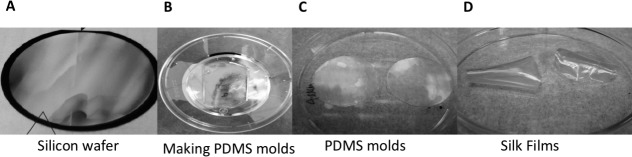
Production of transparent silk films. Photographs illustrating the creation of patterned silk films using soft lithography as described in Material and Methods. (A) Silicon wafer. (B, C) PDMS molds to cast silk solution. (D) Silk films.

Silicon wafers (Fig. 1A) with parallel ridge widths and spacing of 2000, 1000, and 800 nm were produced using standard photolithography techniques as previously described.^6,7^ Polydimethylsiloxane (PDMS) molds were produced from these surfaces by casting 300 μL 10:1 mixture of potting to catalyst solution (Fig. 1B), which was then spread evenly over the entire surface and then degassed for 3 hours to remove air bubble formations. The PDMS was then cured at 60°C for 12 hours, and then the cured PDMS was removed from the patterned silicon surface. The patterned PDMS surfaces (Fig. 1C) were then cut into 35-mm diameter casting surfaces, and then 300 μL 8% silk fibroin solution was pipetted onto each surface. After the silk solution dried into a formed film, the cast films (while still on the PDMS surfaces) were placed in a chamber containing a basin of water beneath a sample shelf. A vacuum of 10 pound per square inch was created, and the films were left to water-anneal for up to 4 hours as previously described.^22^ After processing, the silk films (Fig. 1D) were then removed from their respective PDMS casting surfaces and prepared for cell culture. To produce flat silk film, the exact procedure as stated above was used by casting 300 μL 8% silk fibroin solution pipetted onto a flat PDMS surface.

### Human Corneal Limbal Epithelial (HCLE) Preparation

An HCLE cell line was generously provided by IIene Gipson, PhD, (Schepens Eye Research Institute, Harvard Medical School). Passages 17 to 19 of the HCLE were used. Cells were thawed from storage in liquid nitrogen and cultured for 72 hours in keratinocyte-SFM medium (Gibco, Invitrogen Corporation, Grand Island, NY, USA) supplemented with 0.2 ng/mL mouse epithelial growth factor (EGF; Invitrogen Corporation), bovine pituitary extract (BPE; Invitrogen Corporation), 1% penicillin-streptomycin (P/S; VWR, Radnor, PA, USA), and 0.1% CaCl_2_.2H_2_O (Invitrogen Corporation). A single cell suspension at a density of 1 × 10^4^ cells/cm^2^ were seeded on silk film substrates and cultured for 72 hours before they were collected for further analysis. A seeding density of 1 × 10^4^ cells per cm^2^ was chosen based on the fact cultures would reach near confluency after 3 days post seeding. Culture media was changed every 48 hours post seeding.

### Primary Human Corneal Epithelial Cell (HCEC) Preparation

HCECs were isolated from the human limbal ring of three corneas obtained from the eye bank (Eye Bank for Sight Restoration, Inc., New York, NY, USA). These eyes are from male and female patients aged 50 to 70. After the removal of iris, endothelium, conjunctiva, and the 7-mm central corneal button, the corneoscleral ring was divided into quadrants of 1 × 1 mm in size, placed on a six-well tissue culture plate (VWR) precoated with 50 μg/mL collagen I (BD Biosciences, Bedford, MA, USA) with epithelium side down, and washed in EpiLife medium (Invitrogen Corporation) containing 1% P/S (VWR) and 0.1% Fungizone (Invitrogen Corporation) for 20 minutes. Five hundred microliters of EpiLife medium supplemented with 1% human keratinocyte growth supplement (Invitrogen Corporation), with 1% P/S (VWR), 5% fetal bovine serum (FBS; Thermo Fisher Scientific, Inc., Waltham, MA, USA), 10 mg/mL mouse EGF (Invitrogen Corporation), and 10^−10^ M cholera toxin A (Sigma-Aldrich Corp.) was added to each explant and incubated overnight at 37°C and 5% CO_2_ incubator. The next day, 1 mL complete EpiLife medium was added. Medium was changed every 3 days until 80% confluence was achieved. Cells were then subcultured by washing with Dulbecco's Phosphate-Buffered Saline (DPBS; VWR) twice and digestion with TrypLE Express (Invitrogen Corporation) at 37°C for 12 minutes. A single cell suspension at a density of 1 × 10^4^ cells/cm^2^ were seeded on silk film substrates and cultured for 72 hours before they were collected for further analysis. Culture media was changed every 48 hours post seeding.

### Light Microscopy

Carl Zeiss Axiovision Microscope (Carl Zeiss Microimaging GmbH, Jena, Germany) was used to observe cell cultures daily. Images were taken with an Observer Z1 fluorescent microscope (Carl Zeiss, AG) at 10× objective lenses utilizing a 1.6 Optovar optic. AxioCam HRm digital camera (Carl Zeiss) was used to capture phase contrast images.

### Cell Alignment and Shape Analysis

After 72 hours of culture, phase contrast images were taken to perform cell alignment analyses. Cell cultures were inspected 72 hours after seeding because previous work has shown that cell body alignment and shape have been observed at this time point. Thirty different visual fields of 10× phase contrast images were analyzed each for HCEC and HCLE cultured on TCP, flat, and patterned silk substrates. The number of cells analyzed range from 150 to 300 per treatment. Cells on these images were analyzed using the ImageJ software (http://imagej.nih.gov/ij; provided in the public domain by the National Institutes of Health, Bethesda, MD, USA). The long axis and short axis for each cell were located, and the alignment of each cell was defined at the angle between the long axis of the cell and the pattern groove axis. Cell shape factor was defined as the ratio of the cell major length axis to minor length axis.

### Immunofluorescent Staining

HCECs or HCLE cultured on different silk film substrates were fixed with 4% paraformaldehyde (PFA; Electron Microscopy Sciences, Hatfield, PA, USA) for 15 minutes, then rehydrated in PBS containing 0.5% bovine serum albumin (BSA; Sigma-Aldrich Corp.), and 0.05% nonionic surfactant (Triton-X-100; Sigma-Aldrich Corp.) for 1 hour. After fixation, 50 μL primary antibodies (1:600 antivinculin V9131, Sigma-Aldrich Corp.; 1:50 anti-integrin β 1, 12594-1-AP, Proteintech, Rosemont, IL, USA; 1:50 antinesprin 2, GTX121928; GeneTex, Irvine, CA, USA) was incubated with cells overnight at 4°C, followed by incubation with Oregon Green 488 goat antimouse secondary antibody (O11033; Invitrogen Corporation) or Cy3 goat antirabbit secondary antibody (711165151; Jackson Immunoresearch, West Grove, PA, USA) at a 1:500 dilution for 1 hour. F-actin and nuclei were then stained by incubating cells in 1:100 dilution of Alexa Fluor 568 Phalloidin (A12380; Invitrogen Corporation), and 1:10,000 dilution of 4′,6-diamidino-2-phenylindole (DAPI) (83210, AnaSpec, San Jose, CA, USA) for 20 minutes and 5 minutes, respectively, while protected from light. After rinsing using PBS, samples were mounted, and protected with a glass cover slip. Fluorescent staining was visualized using Observer Z1 confocal fluorescent microscope (Carl Zeiss, AG) with both a 10× and 63× objective lenses. An AxioCam HRm digital camera (Carl Zeiss) and AxioVision 4.0 software were used to capture single and z-stack images (45- to 60-layer range) at 0.25-μm slices using DAPI, GFP, and Texas Red filter channels. Deconvolution was performed on each z-stack using 3D Huygens Deconvolution Software (Scientific Volume Imaging BV, The Netherlands). Images for each replicate were taken at the same threshold level. Total fluorescence measurements were performed with Image J software (National Institutes of Health).

### RNA Isolation

HCECs were cultured on tissue culture plastic (TCP), flat silk films, and patterned silk films. A seeding density of 10,000 cells per cm^2^ was used, and the cultures reached near confluency after 3 days post seeding. After 72 hours of incubation, total RNA was extracted using Qiagen RNeasy Plus Mini Kit (Qiagen, Valencia, CA, USA), and RNA integrity and quantity were checked using Agilent Technologies 2100 Bioanalyzer and Nanodrop Spectrophotometer at the Genomics Resources Core Facility of Weill Cornell Medical College.

### NGS Library Preparation and Illumina Sequencing

Total RNA samples (at least 100 ng total RNA per sample) with a minimum RNA integrity number of 8 were sent to the Genomics Resources Core Facility of Weill Cornell Medical College. Total RNA was used with the TruSeq mRNA-seq Sample Preparation Kit (Illumina, San Diego, CA, USA) to construct cDNA libraries, and cDNA libraries were multiplexed with three samples per lane and loaded onto flow cell lanes. Sequencing-by-synthesis of 51-nucleotide length was performed on Illumina Hiseq 2500 sequencing system (Illumina, Inc., San Diego, CA, USA).

### SeqMan NGen Assembler Short Read Aligner Analysis Workflow

SeqMan NGen assembler (DNAstar version 2016, Madison, WI, USA) was used to align RNA-seq reads against the human reference genome GRCh38. Finished assembly was loaded into DNAstar Qseq to compute raw reads per kilobase of exon model per million mapped (RPKM) reads normalized expression values of the transcript isoforms. A stringent filtering criterion of RPKM value 1.0 in at least one sample was used to obtain expressed transcripts. ANOVA was then performed on the log-transformed data to generate fold change between samples collected from different experimental conditions and the *P* value for each transcript. Differentially expressed mRNA isoforms were filtered for a log-fold-change cutoff >1 and *P* value cutoff of 0.05. Gene ontology, heatmaps, and scatter plots were generated using DNAstar Qseq Software. *P* values for gene ontology were calculated using Benjamini Hochberg correction.

### Pathway Analysis

A data set containing differentially expressed gene identifiers and corresponding expression values was uploaded into the Ingenuity Pathways Analysis (IPA) software (Redwood, CA, USA). The application was used to analyze significant pathways and networks. Differentially expressed genes were overlaid onto a global molecular network developed from information contained in the Ingenuity Pathway Knowledge Base (IPKB) that was derived from known functions and interactions of genes published in the literature.

### Quantitative Real-Time PCR (qPCR)

qPCR experiments were performed on HCECs cultured for 3 days and then 14 days on nano-patterned silk films, flat silk film, and TCP (*n* = 3). Fourteen days were chosen arbitrarily to assess relative long term genetic changes. Two hundred nanogram samples of RNA isolated from cultured HCECs were reverse transcribed into cDNA using the Invitrogen High Capacity RNA-to-cDNA Kit (Life Technologies, Grand Island, NY, USA). cDNA samples were diluted to 500 μL (25-fold dilution). Primers were made from Life Technologies. See the Table for primer information. qPCR was performed on cDNA samples using Invitrogen Power SYBR Green Master Mix and pre-made primers according to manufacturer's protocol (Life Technologies). The expressions of candidate genes were normalized against the housekeeping gene, glyceraldehyde-3-phosphate-dehydrogenase (GAPDH; Life Technologies). Differential expression analysis was performed using the ddCt method.^24^

### Statistical Analysis

ANOVA was used to analyze the statistically significance of alignment and qPCR data (StatPlus, Version 6, AnalystSoft, Inc., Walnut, CA, USA). Test results were reported as *P* values with *P* < 0.05 considered as statistically significant.

## Results

### Patterned Silk Films Change HCEC and HCLE Cell Alignment and Morphology

**Figure 2:**
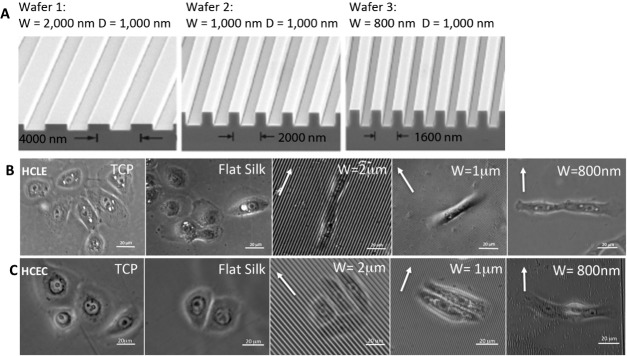
Morphology of HCLE and HCEC on different substrates. HCLE cells and HCECs were grown on TCP, flat silk films, or silk films with different parallel lines pitch. (A) Electron microscopy images of silicon wafers with micro- and nanoscale patterns used to produce patterned silk films. Phase contrast images of HCLE (B) and HCEC (C) cells on different substrate shows that the shape and alignment of the cells changes when they are seeded on patterned silk films. The cells orientate either parallel or perpendicular to the topography indicated by white arrows. W, width; D, depth.

Silk film substrates measuring 50 μm in thickness and having a diameter of 35 mm were successfully produced with varying topographies, including flat and patterned silk films with feature widths of 2 μm, 1 μm, and 800 nm with 1 μm groove depths, respectively. Patterns with parallel ridges and grooves were chosen because focal adhesion numbers and sizes were shown to be increased on parallel ridge patterned silk films.^25^ SEM images of silicon wafers with micro- and nanoscale patterns demonstrated that the desired dimensions were replicated on all silicon surfaces (Fig. 2A).

Primary HCEC and HCLE cells were cultured onto flat and each of the patterned silk film substrates. The experiment was repeated three times to produce triplicate samples. After 72 hours of culture, phase contrast images were taken to perform cell alignment analyses (Figs. 2B, 2C).

**Figure 3:**
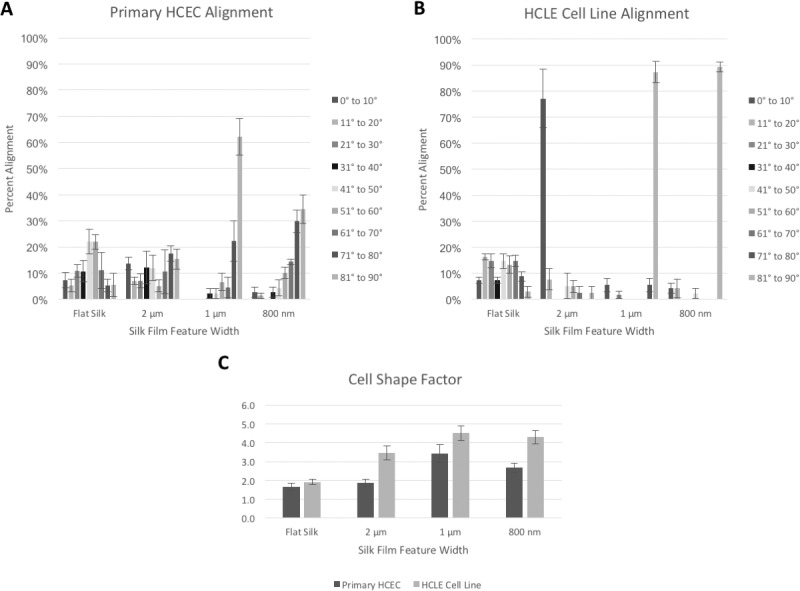
Patterned silk films influence alignment and cell shape. (A, B) HCEC and HCLE cell alignment to the surface pattern (n > 50). Cells aligned either parallel on 2000 nm or perpendicular on 1000 and 800 nm feature width dimensions. (C) Graph showing cell elongation as indicated by increased cell shape factors (ratio of cell major axis over cell minor axis) on patterned surfaces in comparison to flat silk surfaces.

Significant directional cell alignment to the patterns either in the parallel (2 μm) or perpendicular (1 μm and 800 nm) direction of the patterned feature edge axis was found as dependent on the feature width (Figs. 3A, 3B). The greatest cell alignment was found on the 1-μm pattern for both HCEC and HCLE (64% and 89%, respectively, *n* > 50) where cells aligned perpendicular to the feature edge axis. Normal distributions of alignment angles were observed for HCEC and HCLE cultured on flat silk. Cells were more elongated on patterned silk films as indicated by measuring the cell shape factor (ratio of the cell major length axis to minor length axis). The cell shape factor was found to be nearly twofold greater (*P* < 0.001) on the 1-μm patterned surface when compared to the flat silk film surface (Fig. 3C). In addition, HCLE cells aligned to a greater extent when compared to HCEC cells on the patterned silk surfaces.

### Patterned Silk Films Alters HCEC Actin and Vinculin Distribution

**Figure 4:**
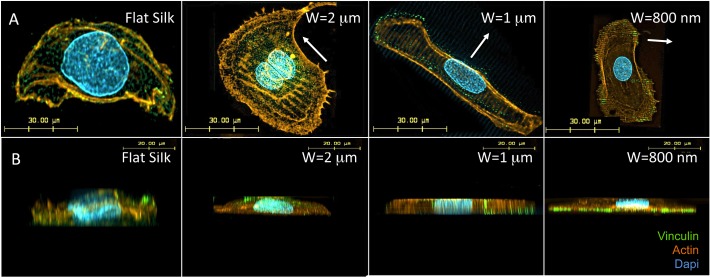
Patterned silk films influence actin alignment and focal adhesions. (A) Confocal microscopy of cell cytoskeletal structure of HCECs. (B) Z-stack projections. The alignment of actin fibers and focal adhesions along patterns were observed.

Figure 4A demonstrates confocal microscopy images of actin fibril formation. It appears that actin fibril alignment occurred along silk film grooves on patterned silk films. Vinculin staining (Fig. 4A) illustrates diffuse vinculin distribution in cells cultured on flat silk films. However, on micro- and nano-patterned silk films, vinculin co-localized with actin filaments and aligned along the pattern feature edges. Z-stack projections illustrate diffuse distribution of actin and vinculin on flat silk films (Fig. 4B). In comparison, on patterned silk films, actin distribution appeared more organized and was observed in both apical and basal cellular surface, while an increased vinculin distribution to the cellular basal surface was observed as the pattern feature widths decreased from micro- to the nanoscale patterns (Fig. 4B).

### Sequencing Run Summary and SeqMan NGen Assembler

**Figure 5:**
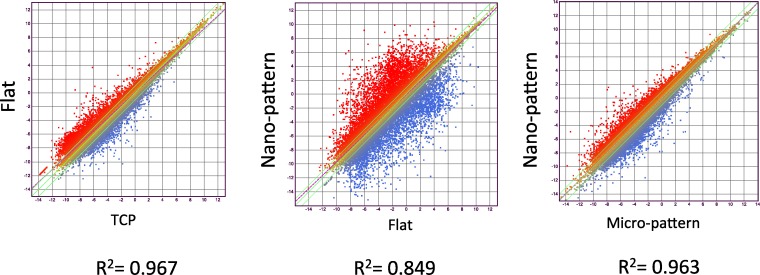
Silk films cause differential gene expression of human corneal epithelial cells. Scatter plots of RNA-Seq results comparing genetic expression profile of HCLE cells cultured on TCP versus flat silk film, flat silk film versus nano-patterned silk film, and micro-patterned silk film versus nano-patterned silk films at 3 days.

**Figure 6:**
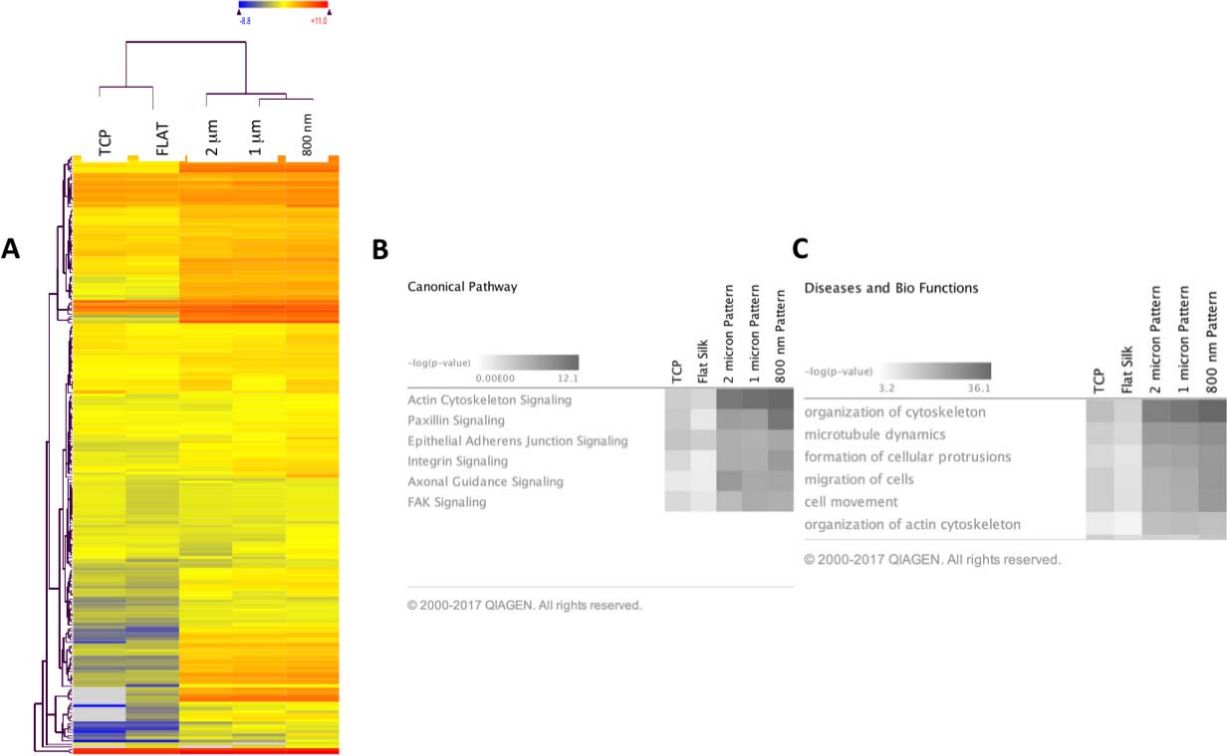
Silk films cause differential expression of genes involved in cytoskeletal dynamics. (A) Heat map representing the average expression values (RPKM) of differentially expressed cell cytoskeletal genes for each of the five experimental groups (primary HCECs cultured on TCP, flat silk, and 2000, 1000, and 800 nm patterned silk film) and hierarchical clustering of groups. (B) Differentially expressed genes for HCECs cultured on each patterned silk substrate in comparison to flat silk are analyzed using IPA. IPA analysis demonstrating the top signaling pathways involved and the corresponding P values. (C) IPA analysis demonstrating top bio functions involved.

A total of 15 libraries of primary HCEC cDNA were sequenced from cultures grown on TCP, flat silk film, and patterned silk films with topographical features of parallel ridge widths of 2 μm, 1 μm, and 800 nm to obtain approximately 50 to 60 million raw sequence reads per sample (*n* = 3). Based on QSeq analysis workflow, 20,984 transcripts were detected with a normalized RPKM value greater than 1.0 in all 15 samples. Of the 20,984 transcripts, 388 genes related to cell cytoskeletal structures met our criteria of differential expression (FC > 1.2, *P* < 0.05). Figure 5 illustrates scatter plots of RNA-Seq results comparing gene expression profile of HCEC cultured on TCP versus flat silk film, flat silk film versus nano-patterned silk film, and micro-patterned silk film versus nano-patterned silk films at 3 days of culture. This figure illustrates a large number of differentially expressed genes were found comparing cells cultured on flat and nano-patterned silk films. *R*^2^ value is smaller for the flat versus nano-pattern scatter plot in comparison to TCP versus flat and micro- versus nano-pattern. Figure 6A illustrates a heat map of the average value of differentially expressed gene transcript for each group. It was observed that the direction of changes in gene expression pattern on all patterned silk film substrates were similar. However, the degree of changes in gene expression is greater on the nano-patterned silk film surface when compared to all other surfaces.

### Patterned Silk Films Alter Genes Involved in Cellular Adhesion and Cytoskeletal Dynamics Related Pathways

IPA was used to analyze the functional importance of differentially expressed genes in cells cultured on patterned silk in comparison to flat silk. The possible functional relationships among differentially expressed genes involved in cell cytoskeletal dynamics were studied. The top canonical pathways that showed significant changes according to IPKB analysis include paxillin signaling, actin cytoskeletal signaling, integrin signaling, and epithelial adherence junction signaling, and focal adhesion kinase (FAK) signaling (Fig. 6B). The differentially expressed genes also play major bio-functions. IPA analysis demonstrates that the top bio-functions involved are organization of cytoskeleton, microtubule dynamics, formation of cellular protrusions, migration of cells, and organization of actin cytoskeleton (Fig. 6C). Table shows major genes and their expression values (RPKM values) involved in each signaling pathway.

### qPCR Revealed Differentially Expressed Genes Involved in Cytoskeletal Dynamics-Related Pathways

**Figure 7:**
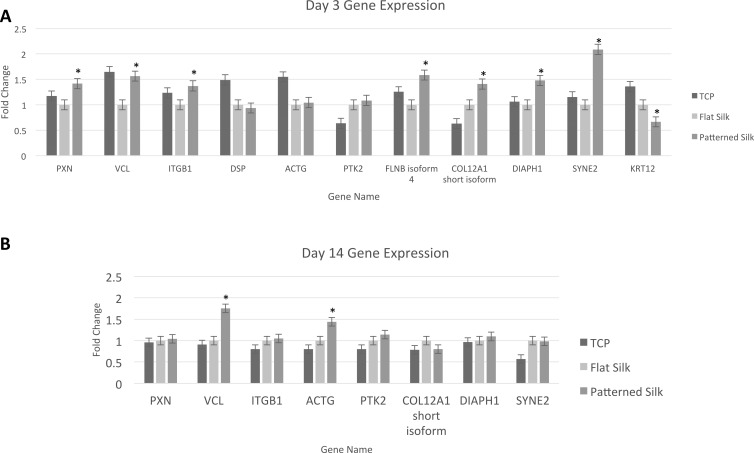
qPCR validation of selected genes involved in cytoskeletal dynamics. qPCR verification illustrating fold change of different genes expressed by HCEC cultured on TCP, flat silk film, and nano-patterned silk film after 3 days (A) and 14 days (B) of culture. PXN, paxillin; VCL, vinculin; ITGB1, integrin β 1; DSP, desmoplakin; ACTG, γ actin; PTK2, protein tyrosine kinase 2; FLNB, filamin B; COL12A1, collagen type XII α 1; DIAPH1, diaphanous-related formin; SYNE2, nesprin 2; KRT12, keratin 12. *P < 0.05.

The RNA-Seq results were confirmed by qPCR of select cytoskeletal-related genes. As shown in Figure 7A, after 72 hours in culture, primary HCECs exposed to silk with nanopatterned surfaces (800 nm pattern), expressed higher levels of PXN, ITGB1, and VCL in comparison to HCECs cultured on flat silk films among many other genes involved in cytoskeletal dynamics-related pathways (*P* < 0.001). Although, not statistically significant, qPCR also showed greater expression of PTK2 (gene coding for FAK gene), and ACTG on patterned silk in comparison to flat silk. No significant change in the expression of CD44 (another cell-surface glycoprotein involved in cell–cell interactions) was found in either RNA-Seq or qPCR (data not shown).

Primary HCECs were then cultured for 14 days on silk film substrates to examine the gene expression of cells exposed to nano-patterned surfaces (Fig. 7B). qPCR of select cytoskeletal-related genes showed increased expression of VCL and ACTG on patterned silk (*P* < 0.01 and *P* < 0.05, respectively). No statistically significant changes in the expression of ITGB1, PXN, and PTK2 were found.

The results of qPCR were compared to fold changes found using RNA-Seq. It was found that good correlation (*R*^2^ = 0.602) exists between the two analyses. Thus, this analysis provides validation of the results of study.

### Immunofluorescence Staining Revealed Differentially Expressed Proteins Involved in Cytoskeletal Dynamics-Related Pathways

**Figure 8:**
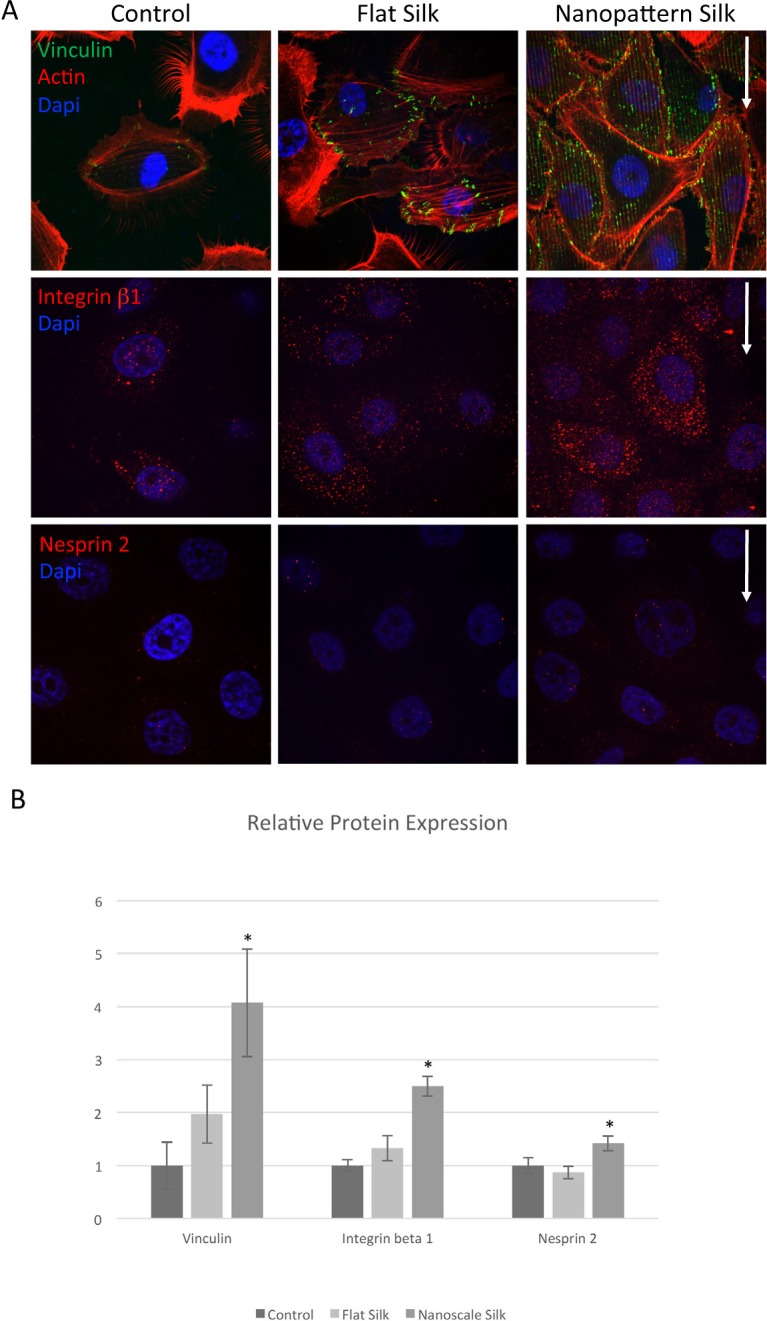
Patterned silk films lead to increased expression of proteins involved in cytoskeletal dynamics. (A) Immunofluorescence staining of HCLE cells cultured for 3 days on TCP, flat silk, and nano-patterned silk films demonstrating expressions of vinculin (green), integrin β 1 (red), and nesprin 2 (red). (B) Relative protein expression demonstrating increased expression of vinculin, integrin β1, and nesprin 2 on nano-patterned silk films (P < 0.05).

Immunofluorescence staining was then performed on human corneal epithelial cells cultured on different cell substrates. Figure 8A demonstrates the expression of vinculin, a membrane-cytoskeletal protein involved in focal adhesion plaques, integrin β1, part of a membrane receptor involved in cell adhesions, and nesprin 2, which links the nuclear envelope to the cytoskeleton and provides mechanosensory functions in cells. Figure 8B demonstrates their relative protein expression, and it appears that significantly higher expressions were found on the nano-pattern silk films.

**Table:**
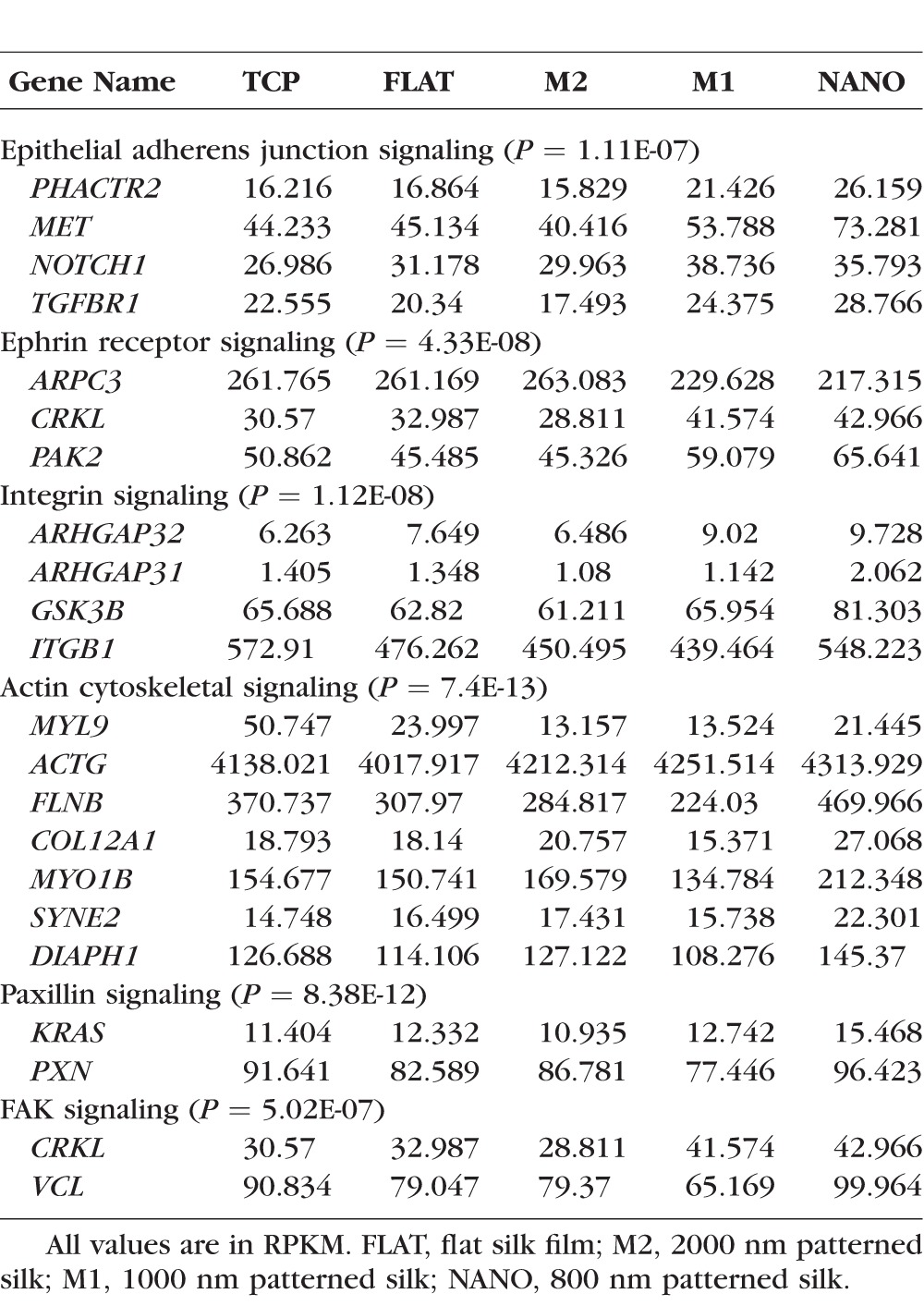
RNA-Seq Result of HCEC Cultured on Different Silk Substrates and TCP Showing Significantly Changed Genes From Each Signaling Pathway and Their Corresponding RPKM Values

## Discussion

Adhesion-mediated sensing of the local cellular microenvironment is a widespread phenomenon that has been demonstrated in various cell types and various synthetic materials including gold nano-particles, calcite crystals, etc.^13,26–28^ In this study, the ability of human corneal epithelial cells to sense surface topography was addressed with the primary intent of engineering a surface biomaterial suitable for ocular surface reconstruction. It was demonstrated that micro- and nanoscale surface topographies on silk films can provide biophysical cues that alter cell morphology and cytoskeletal dynamics. These cues can further lead to changes in cell adhesion-mediated gene expression and activate related signaling pathways. Identifying the genetic basis for these physical cues and the specific genes and pathways involved may provide targets that can help us to alter cellular properties in the future.

Previously, it was reported that human corneal epithelial cell alignment in EpiLife medium was dependent on patterned topography pitch.^9^ Cells changed their body alignment from perpendicular to parallel directions to the topography edge when the pitch increased from 400 to 4000 nm.^9^ Similarly, in this study the majority of cells aligned perpendicularly to the topographical feature edges on the smallest of the nanoscale patterns, while more cells aligned parallel to the patterned topography edge on larger microscale features. Overall, the percent of cell alignment either perpendicular or parallel to the topography edge and the degree of change in cell shape were greater on smaller sized patterns and also for HCLE cells when compared to primary HCECs. This difference in cell alignment can be attributed to the greater heterogeneity of primary HCECs such that there may be cells at different stages of differentiation and thus having different levels of responsiveness to topographical cues. In addition, genetic differences among the three donors may also contribute to this heterogeneity.

To gain a better understanding of the ways in which silk surface topography can lead to changes in cell morphology and function, gene expression studies were conducted using RNA-Seq. Our RNA-Seq data demonstrated that although the alignment of corneal epithelial cells differed depending on the size of the surface patterns, the overall change in gene expression was similar for the pattern widths ranging from 800 nm to 2 μm when in comparison to flat silk. However, the degree of change in gene expression was the greatest on the smallest 800 nm feature widths. It is possible that the 800-nm ridges best mimic the ridges found on corneal basement membrane. Previously, it was shown that cells are able to respond to the surrounding ECM properties, which include rigidity, orientation, and topography, through “mechano-sensing.”^12–15,29–33^ Different types of cell surface receptors has been demonstrated to interact with the ECM, including a variety of integrin receptors, stretch-sensitive ion channels, GPCR, tyrosine kinase receptors, RHAMM, CD44, etc.^12–15,30–33^ In various studies, integrin receptor mediated cellular adhesion to the ECM was shown to be crucial for the maintenance of epithelial cell function.^16,33,34^ In our study, cells cultured on patterned silk film substrates showed the greatest changes in gene expression in signaling pathway-related genes involving actin cytoskeletal, paxillin, integrin, and FAK. Our pathway analysis of the RNA-Seq data points to the role of integrin receptors in sensing micro- and nano-patterned silk films. Furthermore, qPCR data and immunofluorescence staining provided validation for the role of the integrin receptor in cellular response to the presence of the silk film topography, as a significant increase in expression of integrin β1 protein at day 3 in cells cultured on patterned silk films was found.

**Figure 9:**
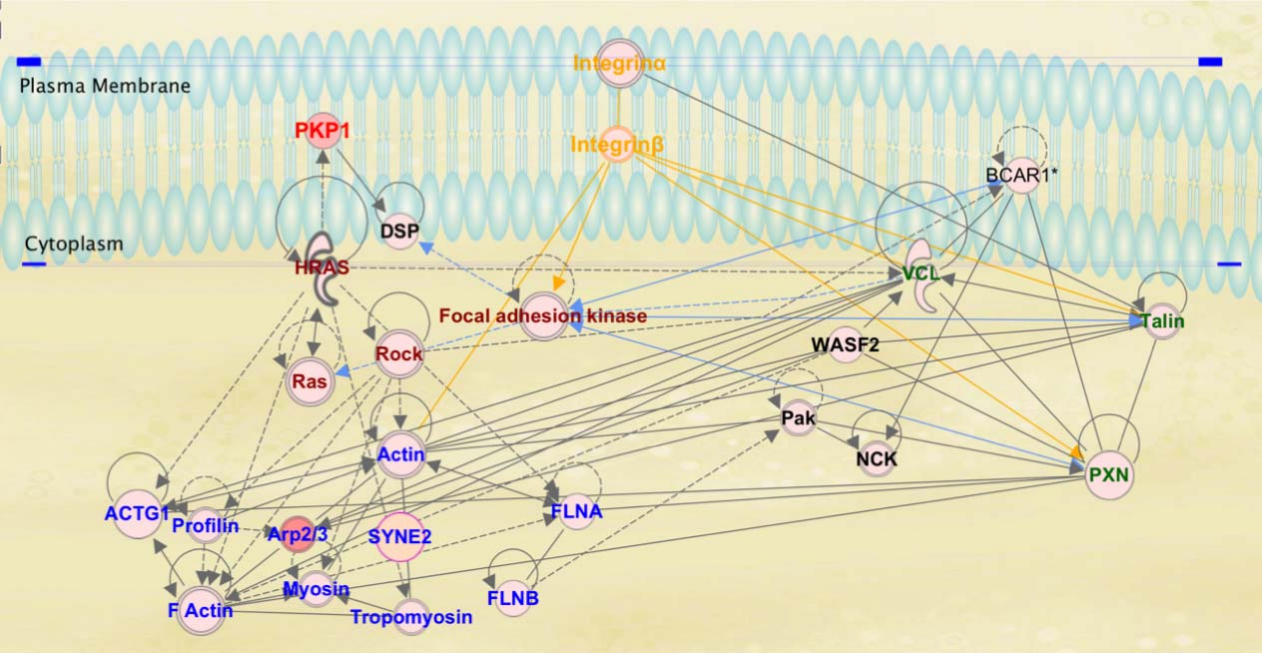
IPKB analysis of relationships among upregulated genes on micro- and nano-scaled silk film topography. (1) Cell surface integrin receptors sense biophysical cues provided by micro- and nano-scale topographical patterns on silk (orange texts); (2) activation of integrin receptor-mediated signaling leads to integrin receptor aggregation and the recruitment and clustering of paxillin, vinculin, talin, and various others involved in focal adhesion formation (green texts); (3) the local clustering of integrins and focal adhesion complex further leads to the recruitment of FAK that initiates a cascade of various signaling events globally (dark red texts); (4) FAK signaling leads to the activation of Ras subfamily of small GTPase (which activates PKP1), the activation of downstream Rho-associated coiled-coil kinases (ROCK), and other key regulators of actin remodeling (dark red texts); (5) global activation of adhesion-mediated signaling pathways induces actin cytoskeletal remodeling (blue texts); and (6) changes in the regulation of gene expression.

Our proposed pathway of mechanotransduction by micro- and nanoscale topographies on silk is demonstrated by our IPKB analysis of relationships among upregulated genes on micro- and nanoscale patterned silk films (Fig. 9). In this pathway analysis, we propose that cell surface integrin receptors sense biophysical cues provided by micro- and nano-scale topographical patterns on silk. It is known that in the process of adhesion to the ECM, clustering of integrin receptors to the site of cell attachment and the recruitment of various structural and signaling molecules to these focal adhesion regions occur.^32,35–37^ This clustering leads to the formation of focal adhesion molecular complexes consisting of various integrin receptors, vinculin, paxillin, and talin.^38–41^ The activation of integrin receptors can also lead to the recruitment of FAK to focal adhesion complex and the activation of downstream signaling molecules, such as Ras and MAP kinases that control actin reorganization, genetic regulation, and various other cellular functions.^32^ Similar to cellular interaction with the ECM, RNA-Seq data in this study suggests that epithelial cell interaction with micro- and nanoscale topographies on silk films change the expressions of genes involved in integrin-mediated pathways, paxillin signaling, and FAK signaling pathways. Micro- and nanoscale surface patterning on silk also lead to the formation of focal adhesion points along pattern and the reorganization of actin cytoskeleton along topographical features as demonstrated in our immunofluorescence staining studies. It was also reported in a previous study conducted by our group that the alignment of actin fibers and focal adhesions along the silk film topography was observed as early as 2 hours after incubation (Pan Z, et al. *IOVS* 2011;52:ARVO Abstract 1944). As mRNA for the current study was collected at 72 hours of incubation, we propose that the change seen in genetic expression occurs after the activation of signaling cascades initiated by integrin-mediated cell adhesion to the silk film topography.

To examine the gene expression pattern of primary HCECs exposed to nanoscale silk film substrates in the long term, cells were cultured for 14 days. qPCR of select cytoskeletal-related genes showed no statistically significant changes in the expression of ITGB1, PXN, but continued increase in the expression of VCL and ACTG on patterned silk surface. This finding suggests that in more long-term cultures, the initial recruitment of ITGB1 upon cell attachment to the surface pattern maybe dampened, as the cell remains attached, migrates, and proliferates. However, actin re-organization and continued adhesion mediated downstream signaling continue to occur.

Our data indicate that micro- and nanoscale silk film topographies can act to mimic aspects of cell-ECM interactions by changing the expression of genes related to signaling pathways activated by cell adhesion to the ECM. Understanding the influence of how different micro- and nanoscale patterns can influence cell behavior and gene expression can aid in the intelligent design of biomaterial surfaces for ocular surface reconstruction and other related clinical applications.
